# An Unsupervised Condition Monitoring System for Electrode Milling Problems in the Resistance Welding Process

**DOI:** 10.3390/s22124311

**Published:** 2022-06-07

**Authors:** Daniel Ibáñez, Eduardo Garcia, Jesús Soret, Julio Martos

**Affiliations:** 1 Department of Electronic Engineering, Universidad de Valencia, Campus de Burjassot, 46100 Burjassot, Spain; jesus.soret@uv.es (J.S.); julio.martos@uv.es (J.M.); 2 Ford Spain, Poligono Industrial Ford S/N, 46440 Almussafes, Spain; egarci75@ford.com

**Keywords:** resistance spot welding, electrode wear, condition monitoring, milling machine, unsupervised clustering

## Abstract

Resistance spot welding is one of the most widely used metal joining processes in the manufacturing industry, used for structural body manufacturing, railway vehicle construction, electronics manufacturing, battery manufacturing, etc. Due to its wide use, the quality of welded joints is of great importance to the manufacturing industry, as it is critical for ensuring the integrity of finished products, such as car bodies, that withstand high levels of stress. The quality of the welding is influenced both by the programming of the welding and by the good condition of the mechanical part that carries out the welding. These mechanical factors, such as electrode geometry and wear, degrade over time. As the welding points are made, the geometry and properties of the electrodes change, so they undergo a milling process to remove impurities and return them to their initial geometry. Sometimes the milling is deficient, and the electrode continues to wear, causing welding problems such as loose spots and metal spatter. This article presents a method for condition monitoring of the milling process and weld wear based on existing data in real production lines. The use of unsupervised clustering methods is proposed to perform a check by which, using current and resistance data, the electrode wear is grouped. Specifically, a method using multidimensional k-means for the condition monitoring of electrode wear is established. This research gives a real and applicable solution for reducing the quality problems caused by milling defects and electrode wear in the production lines of high-production manufacturing industries, presenting a system for sending alarms based on the behavior of welding variables.

## 1. Introduction

Resistance spot welding is one of the most important joining processes in the metallurgy industry due to its efficiency and suitability for automation [1]. Specifically in the automotive industry, modern auto-body assembly needs 7000 to 12,000 spots of welding, and, thus, resistance welding plays a crucial role, representing approximately 90% of the welded joints carried out for body assembly [2].

The welding process can be summarized very simply; the sheet of metal to be welded is placed between water-cooled electrodes and then heat is obtained by passing a large electric current through them for a short period of time [3].

Although this process can be summarized in a very simple way, in reality, there are many factors that affect the achievement of the desired quality. Many programmable parameters affect weld quality. These parameters are given by Joule’s law and are the welding time, the current and the resistance, which is related to the pressure achieved by the electrodes [4]. These parameters must be configured to achieve the desired quality and stability over time. In addition, several factors play an important role in the quality of the weld, such as voltage fluctuation, the misalignment of electrodes or loss of electrode pressure. The shared characteristic of these factors is that they do not change during the lifetime of the welding electrodes and can be better controlled by a better welding controller or machine maintenance [5].

However, another parameter is inherent to the number of welds performed throughout the life of the electrodes: wear. The wear of the electrodes increases as the number of welds increases, modifying both the electrical and thermomechanical properties between the electrodes and the sheets. There are special cases in which this wear is even more pronounced, in particular, in those cases in which the sheets are coated with zinc or when sealer is used between the sheets to be welded. These special cases tend to stick to the copper electrodes, thus, causing a further increase in wear [6].

The heating of the metal can be described according to Joule’s law, represented in Equation (1), where *Q* is the heat generated during welding by passing a current (*I*) along the metal–metal and metal–electrode resistance (*R*) over a period of time [7]:

(1) $$Q=I^{2}Rt$$

**Figure 1:**
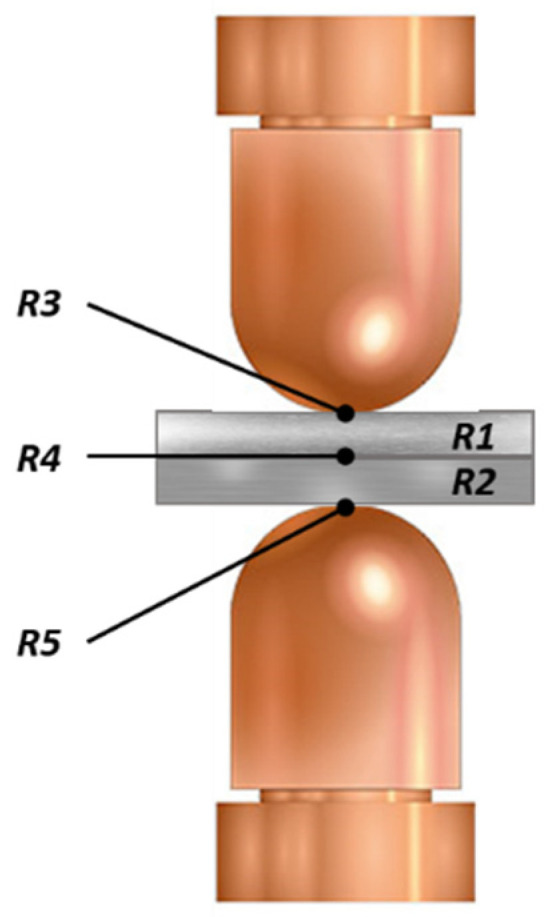
Resistances involved in a resistance spot weld.

In Figure 1, it can be seen schematically, as in resistance spot welding, that three different types of process resistance determine the resistance represented by Joule’s law: contact resistors *R*3, *R*4 and *R*5 between sheet metal resistors *R*1 and *R*2. Contact resistance refers to the resistance generated at the interface between the electrode and the metal sheet (*R*3 and *R*5) and the resistance between the metal sheets (*R*4). The resistance of the sheets is determined by the resistivity and the thickness of the metal. Normally, these resistances are higher than the contact resistance between the electrodes, which causes the fusion to begin at the union of the two metals [8].

From Figure 1, it can also be concluded that good contact between the electrodes and the metal is essential so that the electrode–sheet contact resistance is lower than the metal–metal resistance in such a way that the fusion begins between the metal-to-metal contacts. This bad contact between the electrodes and the metal can be due to different circumstances: misalignment of the electrodes, bad programming of the position of the welding point, dirt on the metals, deformation and wear of the electrodes, etc.

To avoid and correct the wear of the electrodes, a series of milling operations are carried out throughout the electrodes’ useful life. Sometimes, these mills fail to reshape the electrode, leaving dirt in the capsule or leaving the electrode with a different shape to the desired one. This causes the contact resistance to vary, and, in addition, an increase in welding quality problems, such as small, deformed or even non-existent nuggets. Due to the increase in resistance and, therefore, the increase in heat, metal ejections can occur during welding, causing a quality and safety problem in the production line [9].

## 2. Electrode Wear and Milling

Electrode wear is one of the most important issues to research in the resistance welding process. Specifically, studies have focused on determining the factors that influence the appearance of electrode wear. First, Tanaka et al. [10] found that electrode wear could be reduced by increasing the nickel content of the metal foils. In this same line, Rashid et al. [11] demonstrated how a good choice of lubricant coated on the metal sheets could increase the useful life of the electrodes. Similarly, different authors have described the relationship between the different welded materials and the useful life of the electrodes [12,13,14,15,16].

In the same way, the decrease in welding quality caused by the wear of the electrodes has been widely investigated. The reduction in quality is determined by the increase in the diameter of the contact face of the tips [17]. This is because the increase in the diameter of the tip of the electrode, which results in a reduction of the heat generation of the welding joint, causes a decrease in the electrode and is the main reason for the decrease in the size of the nugget [18].

The deposition of the metal coating on the copper electrodes generates a change in the properties of the electrode and, therefore, the wear of the tips [19]. In addition to the reduction of the size of the nugget, the wear of the electrode is of great importance in the presence of weld ejections and other quality defects and can be the cause of 60% of quality problems [20].

Finally, due to the great importance of this defect, different authors have proposed methods for estimating wear or evaluating it. Gauthier et al. [21] and Raoelison et al. [22] demonstrated a method for the numerical modeling of electrode wear which is useful for theoretical estimation but can hardly be applied to real cases where different factors interfere, such as mechanical problems or changes in the production process. Peng et al. [23] proposed the use of images for the analysis of the degradation of the electrodes; the main disadvantage for the application of this system in large production factories is the cost associated with the acquisition of the equipment.

On the other hand, Zhang et al. [24] proposed the use of electrode displacement to determine electrode wear; the discussed method provides a convincing solution but can only be carried out in those welding guns that have an integrated measurement system for electrode displacement, something that is usually lacking in pneumatic welding guns.

Finally, Zhou et al. [25] presented a method based on the analysis of dynamic resistance during welding to determine wear. The main disadvantage of this study is that it assumed that the dynamic resistance trend depends only on the wear of the electrodes when, actually, this variation can depend on different factors, such as the final quality of the welding point, as stated by Zhao et al. [26], or the temperature and pressure, as stated by Whang et al. [27], among many other factors.

**Figure 2:**
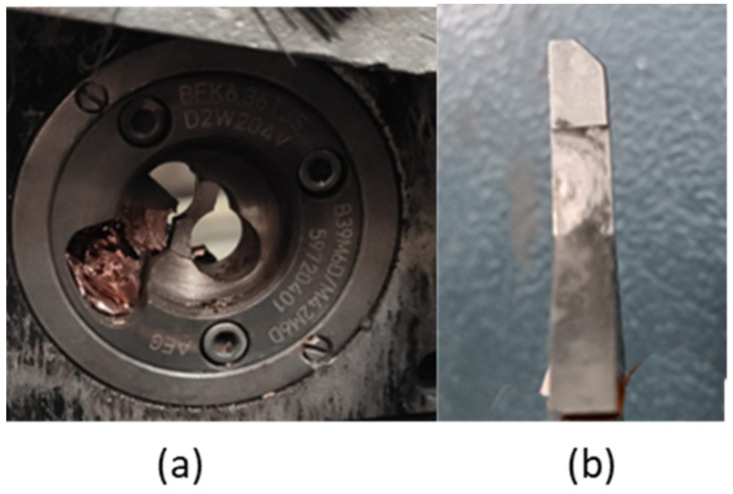
Typical defects of milling machines. (**a**) Milling machine covered by the chips from the electrodes. (**b**) Dull blade.

All these proposed methods were based on the premise that, after performing the milling, the electrodes return to their original geometric state. On multiple occasions, due to mechanical problems of both the welding gun and the milling machine, such as blade wear or issues with milling, as shown in Figure 2, the restoration of the geometry does not occur.

Therefore, the objective of this research is not to propose a system that only determines the wear of the electrode, but one which determines the milling problems that may occur during the production process; that is, the main objective of this research is to avoid the quality problems caused by the wear of the electrodes.

## 3. Materials and Methods

For the creation of the milling problem detection system, it was essential to be able to relate a real variable to an existing defect; this variable had to be acquired and treated to then proceed to the analysis and the elaboration of a data analysis system for evaluation.

Specifically, due to its existing relationship, the use of the measurement of the electrode resistance is proposed for subsequent preprocessing with normalization and feature extraction to later carry out an unsupervised classification method. This allows the setting of detection thresholds based on the behavior of the resistance data.

### 3.1. Electrode Resistance Measurement Method

Electrode wear is one of the essential external factors that determines the stability of weld joints in the resistance welding process.

To avoid these quality problems, after a certain number of welding points, a shaping of the tips is carried out by means of a milling machine. This process can be automatic or manual depending on the type of production line. Sometimes, due to a malfunction of the milling machine, such as an issue with the cutter, a force problem in the welding gun, poor positioning of the milling machine, etc., the electrodes are not well shaped. This is a problem since, until the next milling or replacement of electrodes, they will continue to function with inadequate wear, which can cause serious quality problems.

**Figure 3:**
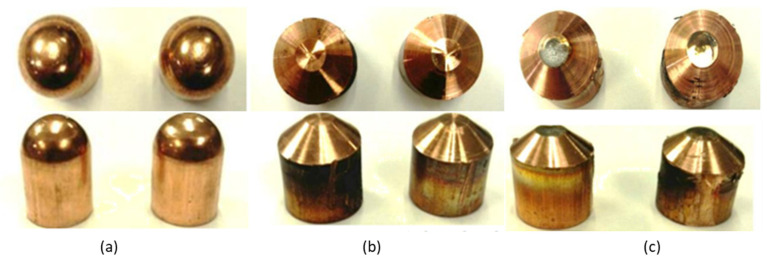
Evolution of the state of the electrodes. (**a**) Type F electrode before milling. (**b**) Type F electrode after good milling. (**c**) F-type electrode after poor milling.

Figure 3 shows the real differences between electrodes after adequate milling and those after defective milling. The electrodes in Figure 3a correspond to 16 mm type F electrodes, according to DIN EN ISO 5821, before being milled for the first time. Figure 3b shows some electrodes that, after executing a certain number of welding points, were milled and returned to their original geometry. Finally, comparing Figure 3b,c, a clear example of poor milling can be seen. In Figure 3c, the active face of the electrodes has been partially cleaned, showing the dirt that generates quality problems.

**Figure 4:**
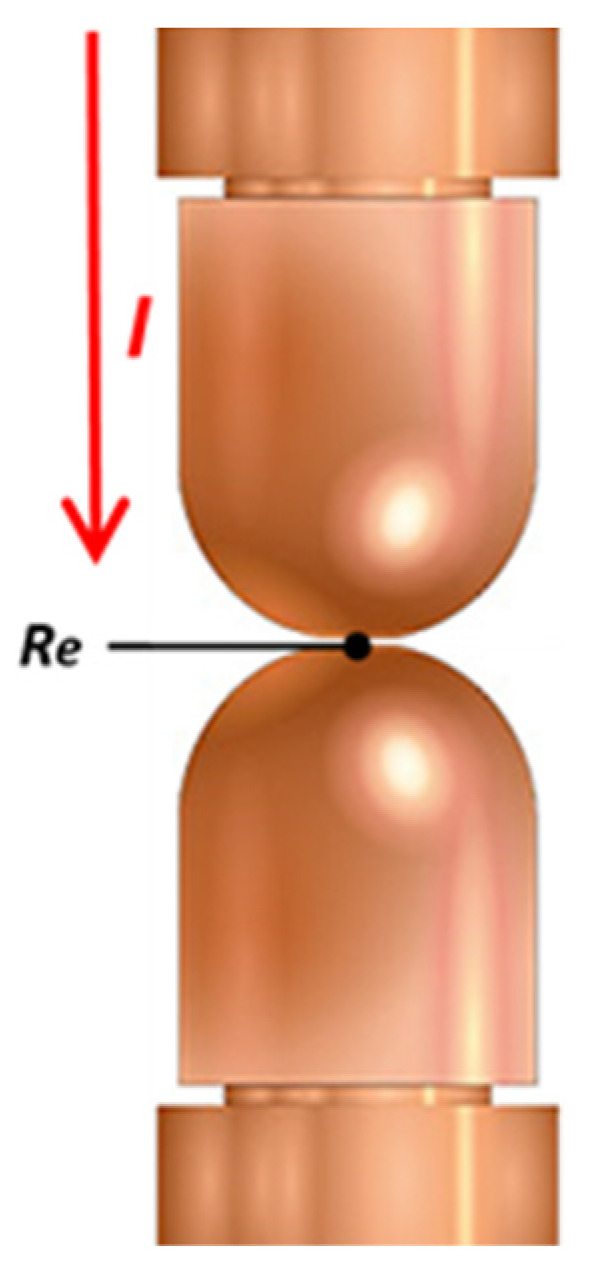
Schema—electrode resistance contact (Re) measurement.

Due to this uncertainty regarding the good milling of the electrodes, a method was established to measure the resistance after each milling is performed, acquiring the voltage and the average current measured between the short-cut electrodes, as shown in Figure 4.

This check is carried out at a constant primary voltage so that when there is a change in the contact resistance of the electrodes (Re) due to the wear of the electrodes, the voltage measured at the electrodes and the current vary according to Ohm’s law.

### 3.2. Data Adquisition

For this article, 650 welding guns located in a real production line were analyzed, as well as a total of 100 millings carried out for each of the electrodes. The welding controls used for the study were BOS6000 with medium-frequency transformers. Regarding the welding guns, the analysis was carried out with no differences between pneumatic guns and servo guns. Similarly, two different welding electrodes were used for the study, 6 mm and 8 mm tip face electrodes, but, at the time of analysis, this difference was considered insignificant for the detection of electrode wear.

In relation to the type of milling machine and electrode, milling machines with an average speed greater than 290 min^−1^ and 25 Nm of torque were used to reset the geometry of the electrode, capable of restoring the geometry of the electrodes according to DIN EN ISO 5821 F1-16-20 [28].

For data acquisition, a pipeline system was implemented between the welding controller and the database through the ELK stack [29]. In this way, a protocol was established to send the welding data to the database every time a welding point occurred, which allowed real-time data analysis, both for machine failure detection and, in this case, for the performance of predictive analysis of weld quality.

**Figure 5:**
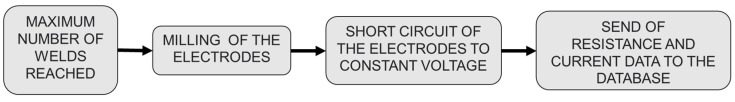
Data acquisition protocol.

For our case, the data acquisition protocol was established, as shown in Figure 5. In the first place, once the maximum number of the welding joints that an electrode could make was reached, keeping the welding quality constant, the electrodes were sent for milling. When the milling was finished, the electrodes were short-circuited by passing a current at constant voltage (PHA method). Finally, the data were stored in the database for further analysis.

### 3.3. Proprocessing and Feature Extraction

Once the necessary programming was carried out in the welding lines, all the data of the 650 welding guns were stored in the database.

**Figure 6:**
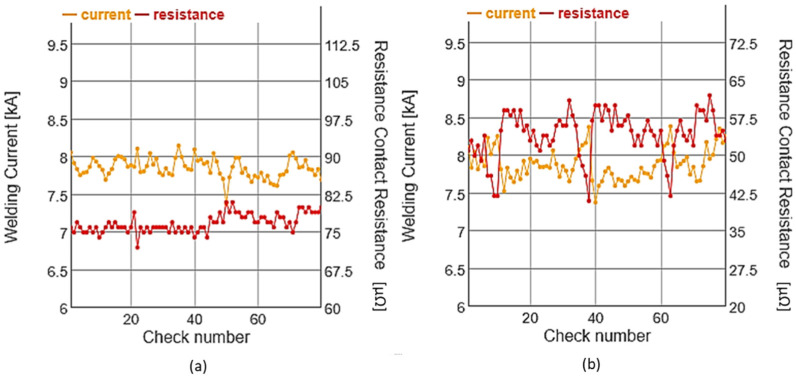
Example of current and resistance data acquired for a weld control. (**a**) Example of the data acquired for a welding gun with correct milling; (**b**) Example of the data acquired for a welding gun with poor milling.

In Figure 6, the data for two different welding guns are shown; it can be seen that the average value of the resistance for each of the cases was different. The difference observed was due to the characteristics of each of the guns, which depended on where the terminals of the voltage probe were located; they affected not only the resistance of the electrodes but also the resistance of the welding arm. The data were always analyzed as normalized data to eliminate this difference from the analysis. Therefore, the z-score normalization, shown in Equation (2), was used. This normalization based on the mean and the standard deviation allowed the reduction of variations if high current and resistance data series were added to the analysis [30].

(2) $$x^{\prime }=\frac{x-\bar{x}}{\sigma }$$

Similarly, in this preprocessing stage, the data were subjected to data cleaning. First, the existing zeros in the time series were removed since those values were meaningless. This is because, when problems appeared when carrying out the check or in the voltage and current measurement probes, a zero was stored in the database. After eliminating the zeros, the atypical data of the time series were calculated, and the outliers were eliminated, for which the expressed formula in Equation (3) was used.

(3) $$\begin{array}{c} Max=Q_{3}+1.5IQR\\ Min=Q_{1}-1.5IQR \end{array}$$

Once this signal was filtered, the feature extraction process was carried out. Feature extraction in machine learning is a process of extracting significant attributes of the data. Feature extraction allows the height of dimensions of a series of data to be reduced to a smaller number of dimensions through unique mapping techniques [31].

For this study, the time series of both resistance and current were reduced to five statistical variables, which allowed the reduction of the dimensions by eighty times for each signal. The calculated variables were:

- The coefficient of variation (CV): the ratio of the standard deviation to the mean;
- Quartile *Q*_1_;
- Quartile *Q*_3_;
- Inter-decile range (IDR): the difference between D9 and D1;
- Median.

As there were two summary signals, in total we had 10 statistical variables as an input dataset for each welding control. The input dataset for the k-mean algorithm was a 650 × 10 array of values. Finally, before proceeding to k-means, the input dataset was standardized to obtain a more precise result in the next section.

### 3.4. K-Means Clustering

In this research, the use of unsupervised clustering using k-means was proposed, Algorithm 1. In general, for this method, the optimal number of clusters for the existing amount of data to be processed is selected first. This parameter represents the number of desired groupings.

**Algorithm 1:** K-means Clustering.**Input:** *X* = {*x*_1_, *x*_2_, ……, *x_n_* } (input data)
*k* (number of clusters)
**Output:**
*C* = {*c*_1_, *c*_2_, ……, *c_k_*} (set of cluster centroids)
**Initialization:**
**for** each *c_i_*
*є C* do:
*c_i_* ← *x_j_ є X* (random selection)
**while:** Convergence or max iteration reached **for** each *x_i_*
*є X* **do**:
minDist ← *minDistance*(*x_i_*, *c_j_*) *j є* (1…*k*);
(based on Euclidian distance $\;\frac{1}{n}\sum (\min j\;d^{2}\;\left(x_{i,}c_{j}\right))$ for *i* = 1 to *n*)
UpdateCluster(*c_i_*)

**Figure 7:**
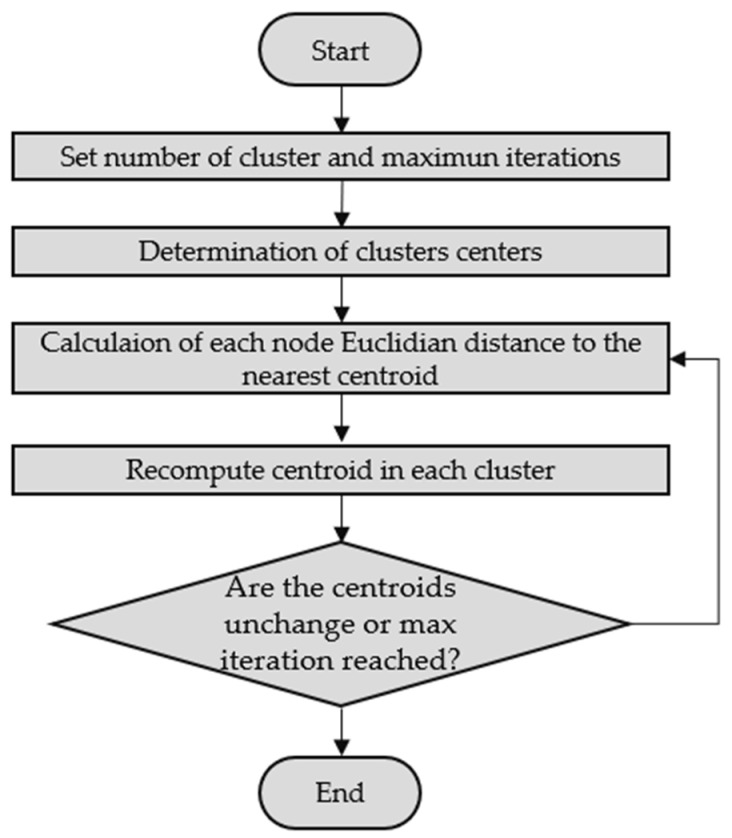
K-means flowchart.

Based on the dataset, the k-means groups them in the programmed number of clusters k, assigning them to the closest centroid. Finally, the algorithm returns both the cluster and the respective centroid. Starting from an initial, non-optimized grouping, the algorithm relocates each point to the nearest new center. It then updates the centers of each cluster based on the mean of the points, repeating this relocation and updating the process until the convergence criteria are satisfied; this process is summarized in the flowchart of Figure 7 [32,33,34].

One of the main advantages of using k-means and unsupervised learning is that it is not necessary to have labeled data. In this study, the population of equipment analyzed was large and varied, which is why it was difficult and inaccurate to label each series of data with the current state of the machine. In this way, it was not necessary to know the characteristics of each of the possible faults that may occur in the milling or in the electrodes, but rather the k-means algorithm, based on the behaviors, assigned each series of data to each cluster.

The purpose of this analysis was to detect any variation in the milling process through its influence on the k-means clustering algorithm. In this case, three different data behavior clusters were expected, and we aimed to establish three machine status criteria: alarm, pre-alarm and good status.

As previously mentioned, the ten statistical variables calculated for the simplification of the model were used as an input dataset for the k-means clustering algorithm. There are different techniques to determine the optimal number of clusters, such as silhouette width, AIC [35] and BIC [36] within the sum of the square (WSS) [37] and NbClust [38]. In this investigation, given that the performance of the AIC and BIC techniques decreases as the dimensionality of the data increases [39] and that the NbClust technique has higher precision than the WSS technique, the optimal number of clusters was identified by the NbClust technique. Specifically, as can be seen in Figure 7, the NbClust function for the input dataset discussed above gave the optimal cluster number for the k-means of the three clusters.

**Figure 8:**
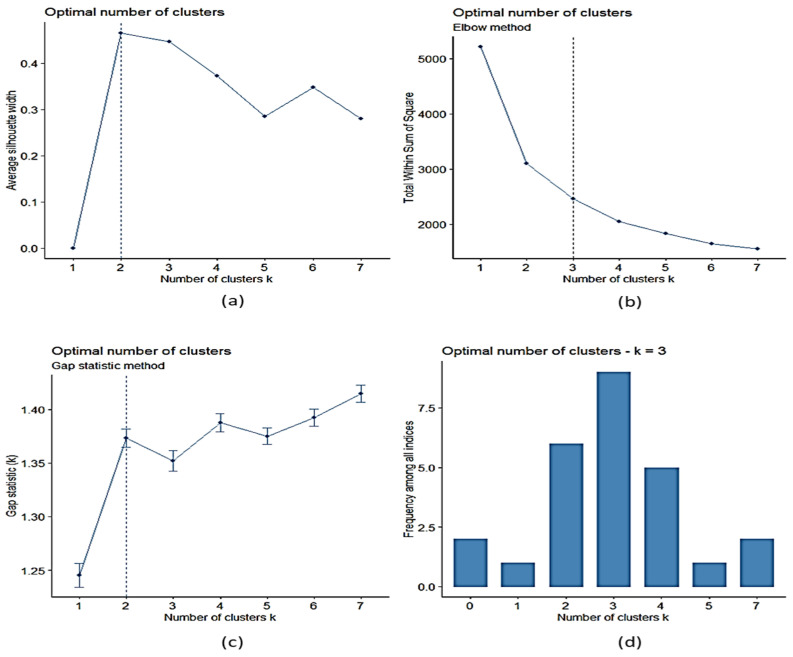
Determination of the optimal number of clusters, (**a**) average silhouette, (**b**) WSS and elbow technique, (**c**) gap statistic method, (**d**) NbClust.

In Figure 8a, the result of the average silhouette technique for choosing the optimal number of clusters is shown; it can be seen that the results for two and three clusters were very close, although the test showed that two was the optimal number of clusters. The same is observable in Figure 8c; although the values of two and three were similar, this technique stated that the optimal choice was two clusters. On the other hand, using the elbow method, as shown in Figure 8b, it was observed that the optimal number of clusters was between three and four clusters. Finally, in Figure 8d, corresponding to the results of the NbClust method, it can be seen that the number of optimal clusters was between two and four, obtaining the highest result for three clusters. Therefore, based on these four analyses and taking into account the greater reliability of the NbClust method, it was established that the optimal number of clusters in this study for the input dataset was three.

## 4. Evaluation and Results

Throughout the previous section, the methodology used, the signals that were analyzed and their dimensional conversion into statistical variables were shown, ending with the method used for clustering and the optimal number of clusters for the proposed dataset.

This section shows the results obtained after using k-means for the grouping of the input dataset. First, the statistical data of each cluster generated were analyzed to determine the behavior corresponding to each cluster.

Table 1 shows the average distance between the points per cluster pair and the distance between the centers of each of the clusters. Several conclusions can be drawn from these values; the distance between centroids was greater between cluster 2 and cluster 3, so cluster 1 could be considered as the central cluster of data deviation, establishing itself as the pre-alarm cluster. Similarly, it was observed that the distance between cluster 1 and cluster 2 was greater than the distance between cluster 2 and cluster 3. This suggests that, due to the dispersion of the data, cluster 2 could be the cluster of points in alarm state.

Table 2 shows the centroids obtained by k-means for each of the input variables; these centroids are the ones that were used to assign the membership of the checks to each cluster and, therefore, their alarm status.

To simplify the cluster plotting process for analysis, these centroids were dimensionally reduced from being ten dimensions, one for each input variable, to two dimensions. These two dimensions were obtained by means of PCA [40]. In Table 2, this reduction in dimensions can be observed with the centroids for dimension 1 (DIM 1) and dimension 2 (DIM 2).

**Figure 9:**
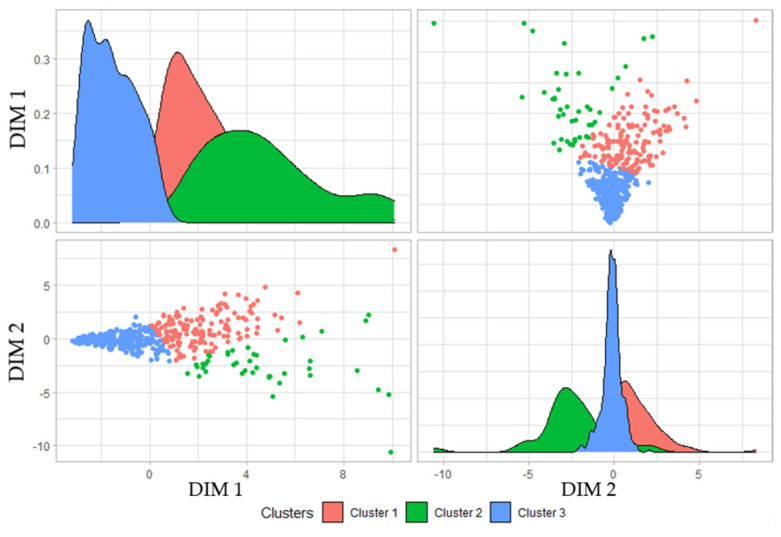
Result with dimensional reduction of the clustering for the input dataset.

To continue with the analysis of the results, the graphs in Figure 9 were analyzed. In this figure, the clusters are represented after their dimensional reduction to two unique dimensions, DIM 1 and DIM2, in order to plot a simpler graph. In the figure, four graphs are represented; two of them show the density distribution for each dimensional reduction. With the help of these two graphs, it can be concluded that, in cluster 3, there were data of those guns with a more stable milling and, therefore, they were correct. This can be confirmed since, observing the distributions of cluster 3 in both dimensions, it can be seen that there was a lower dispersion and a greater normality compared to the other clusters.

In the same way, following the same reasoning as for cluster 3, it was established that cluster 1 represents the millings that begin to be deficient, while cluster 2 groups the deficient millings that start to create quality problems in the welding points due to excessive wear of the electrodes.

**Figure 10:**
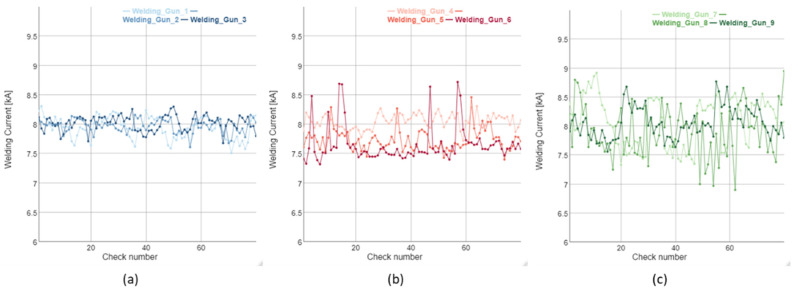
Examples of current measured depending on the cluster for different welding guns. (**a**) Cluster 3, determined as correct operation, (**b**) Cluster 1, determined as pre-alarm, (**c**) cluster 2, determined as alarm.

Finally, Figure 10 shows the current graphs corresponding to each of the clusters. In Figure 10, three current curves grouped in cluster 3 are shown, which correspond to correct operation; if compared with Figure 10a it can be observed that the curves of both graphs have a low dispersion and a stable behavior.

Figure 10b,c shows the current curves for clusters 1 and 2, respectively. From their analysis, it can be concluded that, as the data are assigned from pre-alarm cluster 1 to alarm cluster 2, the curves begin to show greater variance instability, which is an unequivocal sign that the electrodes presented a problem in milling and, therefore, increased wear, which will inevitably turn into quality problems at the weld point.

## 5. Application of the System for Real-Time Detection

This research was not only focused on finding a method that allows the detection of milling problems. The high production rate of manufacturing factories makes it essential that production defects are evaluated in real time. This allows the reduction of costs caused by having to repair products manufactured with poor quality since early detection can reduce the number of poor-quality products manufactured.

The clustering method for the detection of milling problems and electrode wear groups the behavior of the data series in three differentiated clusters: correct operation, pre-alarm and alarm. These three clusters, therefore, allow an algorithm to evaluate and label the status of each of the welding guns in a factory. The real-time detection system is designed to analyze each welding gun in particular and send the operators in charge of that welding line the alarm so that the defect and its possible consequences can be repaired.

As mentioned in the previous sections, a communication structure is necessary between the welding equipment and the database so that the data from all the welding equipment is accessible from the data analysis software. The system for detecting milling problems and electrode wear first collects resistance and current data from each of the welding equipment, labeling those controls that do not have enough data due to communication problems. Next, the extracted data are normalized, as described in the previous sections, and the dimensional reduction of input variables is carried out.

Once the reduction of the time series to the ten input variables has been carried out, the cluster each one of the analyzed pieces of welding equipment belongs to is determined. The assignment of each cluster is carried out by calculating the distance between each point with respect to the centroids of each of the clusters.

The assignment of each of the clusters determined after measuring the distance to each of them allows each piece of the welding equipment to be labeled according to its status in such a way that the welding equipment that is assigned to cluster 3 presents correct operation, and those in cluster 2 are in alarm and, therefore, require corrective action to be carried out.

**Figure 11:**
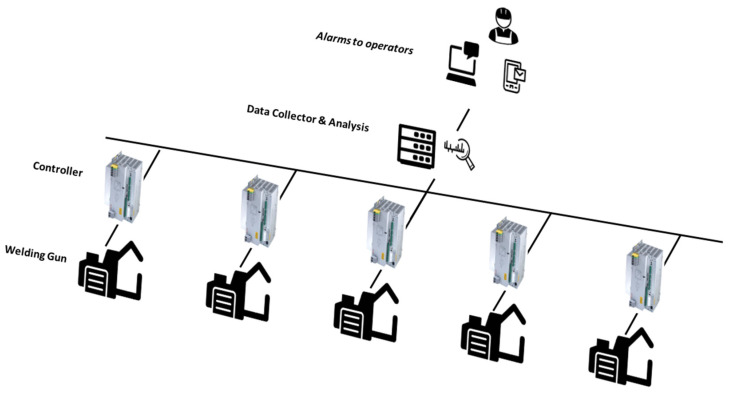
Real-time data collection schema.

Finally, once it has been determined that the welding guns have a behavior typical of electrodes with high wear, the alarm dispatch system is carried out to the production lines. In this case, a publish/subscribe protocol based on AMQP is established [41]. This protocol allows the sending of messages in specific queues. In this case, queues managed by RabbitMQ are used, which allows the sending of alarms through Webex to those in charge of repairing the conflicting equipment. The system is like the one proposed by García and Montes [42] for the acquisition of data from PLC in real time in factories, but, in this case, it is not based on data stored in a PLC but rather the welding equipment itself stores the data through Logstash, making it accessible from the data collector. In short, as described in Figure 11, the proposed system collects data directly from the real production lines and, after data processing, can label defects and send alarms for the repair and correction of quality problems produced.

## 6. Conclusions

This research presents a novel method for the detection of milling problems and electrode wear using unsupervised clustering methods. Throughout this article, the relationship between the serial time data of resistance and the variation of the mechanical properties of the electrodes was described.

Despite working with time series, feature extraction was carried out to reduce dimensionality, which allowed the reduction of the number of input inputs of the clustering algorithm. This also allowed the input data to be scaled so that they were not influenced by the resistance differences existing in each welding gun.

The main advances obtained from this research were the following:

- A method for detecting the relationship between welding variables and milling state;
- An alarm system, where pre-alarm status and correct operation are established according to the output of the clustering algorithm;
- A system for the collection of data in a welding line that allows the realization of data analysis in real time, both for this investigation and for future investigations.

Despite the advances described, the system is still not capable of differentiating between types of fault. Different mechanical factors influence milling problems, such as worn blades, transformer secondary circuit problems, etc. The objective of future work in this investigation should go from the cataloging of faults as alarm, pre-alarm and correct status to a fault labeling system based on behavior pattern. In the same way, throughout this investigation, unsupervised learning methods were used due to the characteristics of the sample, but, in future works, we expect to continue in the line of experimentation with other analysis methods that could improve the detection system.

## Figures and Tables

**Table 1:** Matrix of separation values between all pairs of clusters and distance between centroids.

Cluster	1		2		3	
	Separation	Centroids	Separation	Centroids	Separation	Centroids
1	0.00	0.00	0.98	5.66	0.48	3.11
2	0.98	5.66	0.00	0.00	3.70	8.10
3	0.481	3.11	3.70	8.10	0.00	0.00

**Table 2:** Cluster centroids for each dimension.

Cluster	C CV	C *Q*_1_	R CV	R *Q*_1_	C *Q*_3_	R *Q*_3_	IDR R	C *Q*_2_	R *Q*_2_	IDR C	DIM 1	DIM 2
1	0.71	0.64	0.48	0.65	−0.66	−0.62	0.83	−0.16	0.25	0.82	−1.58	−0.18
2	2.23	2.19	2.11	2.39	−2.39	−2.15	1.96	2.42	−2.61	1.83	1.86	0.67
3	−0.60	−0.55	−0.45	−0.57	0.58	0.54	−0.65	−0.11	0.07	−0.63	6.69	−2.26

## Data Availability

Not applicable.

## References

[1] Hou Z., Kim I.-S., Wang Y., Li C., Chen C. (2007). Finite element analysis for the mechanical features of resistance spot welding process. J. Mater. Process. Technol..

[2] Huh H., Kang W. (1997). Electrothermal analysis of electric resistance spot welding processes by a 3-D finite element method. J. Mater. Process. Technol..

[3] Özyürek D. (2008). An effect of weld current and weld atmosphere on the resistance spot weld ability of 304L austenitic stainless steel. Mater. Des..

[4] Aravinthan A., Nachimani C. (2011). Analysis of Spot Weld Growth on Mild and Stainless Steel. Weld. J..

[5] Tang H., Hou W., Hu S.J., Zhang H.Y., Feng Z., Kimchi M. (2003). Influence of welding machine mechanical characteristics on the resistance spot welding process and weld quality. Weld. J..

[6] Zhang J., Zhang P.X., Xu X.J. (2014). A Model for Predicting the Wear Degree of Electrode Tip. Appl. Mech. Mater..

[7] Jeffus L. (1999). Welding Principals and Applications.

[8] Del Vecchio E.J. (1956). Resistance Welding Manual.

[9] Zhang X., Chen G., Zhang Y. (2008). Characteristics of electrode wear in resistance spot welding dual-phase steels. Mater. Des..

[10] Tanaka Y., Sakaguchi M., Shirasawa H. (1987). Electrode life in resistance spot welding of zinc plated steel sheets. Int. J. Mater. Prod. Technol..

[11] Rashid M., Fukumoto S., Medley J.B., Villafuerte J., Zhou Y. (2007). Influence of lubricants on electrode life in resistance spot welding of aluminum alloys. Weld. J..

[12] Kondo M., Konishi T., Nomura K., Kokawa H. (2013). Degradation mechanism of electrode tip during alternate resistance spot welding of zinc-coated galvannealed and uncoated steel sheets. Weld. Int..

[13] Gould J.E., Kimchi M., Campbell D.H. (1988). Weldability and Electrode Wear Characteristics of Hot-Dip Galvanized Steel with and without a Ferrophos Containing Primer.

[14] Saito T., Takahashi Y., Nishi T. (1988). Electrode Tip Life in Resistance Spot Welding of Zinc and Zinc Alloy Coated Sheet Steels. Nippon. Steel Tech. Rep..

[15] Athi N., Cullen J., Al-Jader M., Wylie S., Al-Shamma’A A., Shaw A., Hyde M. (2009). Experimental and Theoretical Investigations to the Effects of Zinc Coatings and Splash on Electrode Cap Wear. Measurement.

[16] De A., Dorn L., Gupta O. (2000). Analysis and Optimisation of Electrode Life for Conventional and Compound Tip Electrodes During Resistance Spot Welding of Electrogalvanised Steels. Sci. Technol. Weld. Join..

[17] Fukumoto S., Lum I., Biro E., Boomer D.R., Zhou Y. (2003). Effects of electrode degradation on electrode life in resistance spot welding of aluminum alloy 5182. Weld. J..

[18] Wang B., Hua L., Wang X., Song Y., Liu Y. (2015). Effects of electrode tip morphology on resistance spot welding quality of dp590 dual-phase steel. Int. J. Adv. Manuf. Technol..

[19] Chan K.R. (2005). Weldability and Degradation Study of Coated Electrodes for Resistance Spot Welding. Master’s Thesis.

[20] Xia Y.-J., Su Z.-W., Li Y.-B., Zhou L., Shen Y. (2019). Online quantitative evaluation of expulsion in resistance spot welding. J. Manuf. Process..

[21] Gauthier E., Carron D., Rogeon P., Pilvin P., Pouvreau C., Lety T., Primaux F. (2014). Numerical Modeling of Electrode Degradation During Resistance Spot Welding Using CuCrZr Electrodes. J. Mater. Eng. Perform..

[22] Raoelison R., Fuentes A., Pouvreau C., Rogeon P., Carré P., Dechalotte F. (2014). Modeling and numerical simulation of the resistance spot welding of zinc coated steel sheets using rounded tip electrode: Analysis of required conditions. Appl. Math. Model..

[23] Peng J., Fukumoto S., Brown L., Zhou N. (2004). Image analysis of electrode degradation in resistance spot welding of aluminium. Sci. Technol. Weld. Join..

[24] Zhang Y.S., Wang H., Chen G.L., Zhang X.Q. (2007). Monitoring and intelligent control of electrode wear based on a measured electrode displacement curve in resistance spot welding. Meas. Sci. Technol..

[25] Zhou L., Li T., Zheng W., Zhang Z., Lei Z., Wu L., Zhu S., Wang W. (2020). Online monitoring of resistance spot welding electrode wear state based on dynamic resistance. J. Intell. Manuf..

[26] Zhao D., Bezgans Y., Wang Y., Du W., Vdonin N. (2021). Research on the correlation between dynamic resistance and quality estimation of resistance spot welding. Measurement.

[27] Wang S.C., Wei P.-S. (2000). Modeling Dynamic Electrical Resistance During Resistance Spot Welding. J. Heat Transf..

[28] (2009). Resistance Welding—Spot Welding Electrode Caps (ISO 5821:2009).

[29] Michael M., Davvid S. (2016). The Rise of Elastic Stack. https://www.researchgate.net/publication/309732494_The_Rise_of_Elastic_Stack?channel=doi&linkId=5820655c08ae40da2cb4e19a&showFulltext=true.

[30] Mohamad I.B., Usman D. (2013). Standardization and Its Effects on K-Means Clustering Algorithm. Res. J. Appl. Sci. Eng. Technol..

[31] Lakshmanan M., Karnan H., Natarajan S. (2020). Smart Diagnosis of Cardiac Arrhythmias Using Optimal Feature Rank Score Algorithm for Solar Based Energy Storage ECG Acquisition System.

[32] MacQueen J. (1967). Some methods for classification and analysis of multivariate observations. Proceedings of the Fifth Berkeley Symposium on Mathematical Statistics and Probability.

[33] Steinley D. (2006). K-means clustering: A half-century synthesis. Br. J. Math. Stat. Psychol..

[34] Swana E., Doorsamy W. (2021). An Unsupervised Learning Approach to Condition Assessment on a Wound-Rotor Induction Generator. Energies.

[35] Akaike H. (1987). Factor analysis and AIC. Psychometrika.

[36] Burnham K.P., Anderson D.R. (2004). Multimodel inference: Understanding AIC and BIC in model selection. Sociol. Methods Res..

[37] Amruthnath N., Gupta T. Fault Class Prediction in Unsupervised Learning using Model-Based Clustering Approach. Proceedings of the 2018 International Conference on Information and Computer Technologies (ICICT).

[38] Malika C., Ghazzali N., Boiteau V., Niknafs A. (2014). NbClust: An R Package for Determining the Relevant Number of Clusters in a Data Set. J. Stat. Softw..

[39] Broman K.W., Speed T.P. (2002). A model selection approach for the identification of quantitative trait loci in experimental crosses. J. R. Stat. Soc. Ser. B (Stat. Methodol.).

[40] Salem N., Hussein S. (2019). Data dimensional reduction and principal components analysis. Procedia Comput. Sci..

[41] (2006). AMQP: Advanced Message Queuing Protocol, Version 0.8. AMQP Working Group Protocol Specification. https://www.rabbitmq.com/resources/specs/amqp0-8.pdf.

[42] Garcia E., Montes N. (2019). Mini-term, a novel paradigm for fault detection. IFAC-PapersOnLine.

